# Dynamics of the discovery process of protein-protein interactions from low content studies

**DOI:** 10.1186/s12918-015-0173-z

**Published:** 2015-06-06

**Authors:** Zichen Wang, Neil R. Clark, Avi Ma’ayan

**Affiliations:** Department of Pharmacology and Systems Therapeutics, Icahn School of Medicine at Mount Sinai, One Gustave L. Levy Place Box 1215, New York, NY 10029 USA; BD2K-LINCS Data Coordination and Integration Center, New York, USA; Knowledge Management Center for the Illuminating the Druggable Genome project, New York, USA

## Abstract

**Background:**

Thousands of biological and biomedical investigators study of the functional role of single genes and their protein products in normal physiology and in disease. The findings from these studies are reported in research articles that stimulate new research. It is now established that a complex regulatory networks's is controlling human cellular fate, and this community of researchers are continually unraveling this network topology. Attempts to integrate results from such accumulated knowledge resulted in literature-based protein-protein interaction networks (PPINs) and pathway databases. These databases are widely used by the community to analyze new data collected from emerging genome-wide studies with the assumption that the data within these literature-based databases is the ground truth and contain no biases. While suspicion for research focus biases is growing, a concrete proof for it is still missing. It is difficult to prove because the real PPINs are mostly unknown.

**Results:**

Here we analyzed the longitudinal discovery process of literature-based mammalian and yeast PPINs to observe that these networks are discovered non-uniformly. The pattern of discovery is related to a theoretical concept proposed by Kauffman called “expanding the adjacent possible”. We introduce a network discovery model which explicitly includes the space of possibilities in the form of a true underlying PPIN.

**Conclusions:**

Our model strongly suggests that research focus biases exist in the observed discovery dynamics of these networks. In summary, more care should be placed when using PPIN databases for analysis of newly acquired data, and when considering prior knowledge when designing new experiments.

**Electronic supplementary material:**

The online version of this article (doi:10.1186/s12918-015-0173-z) contains supplementary material, which is available to authorized users.

## Background

Protein-protein interaction networks (PPINs) are an abstract representation of the body of knowledge about the known physical interactions between proteins within cells of an organism. In these networks, proteins are the nodes and their known physical interactions (PPIs) are the links. Literature-based PPINs and pathway databases are central in computational systems biology since they summarize accumulated knowledge and are reused for various types of analyses. For example, PPINs can be used to predict disease genes and identify disease related pathways or modules [1–5], applied to predict gene/protein function [6, 7] and predict undiscovered PPIs [8]. Commonly, lists of genes and proteins identified experimentally by high content profiling methods use literature curated PPINs and pathway databases for enrichment analyses [9], or such lists are seeded within PPINs to identify functional subnetworks, and this helps to provide global biological context to the identified gene lists [10, 11]. Inclusion of PPINs was shown to improve the quality of inferred co-expression networks and the prioritization of genes that harbor mutations and copy number variations to better correlate these with disease [12–14].

There are several reasons to suspect that literature-based PPINs and pathway databases contain research focus biases. For instance, the uneven availability of tools such as mouse models or quality antibodies enable the study of some genes and proteins over others [15]. However, so far, concrete proof that such discovery bias really exists has not been reported. It is difficult to prove that such bias exists because the real PPINs are mostly unknown. One null model for the discovery of any network is a uniformly even, uncorrelated exploration of all links and nodes without bias. An alternative model can simulate the network discovery process whereby the discovery in one region of the network will predispose the expansion of related discoveries. Such models can be compared to empirical observations. Tria et al. [16] empirically observed that with open data resources, such as online music catalogues and Wikipedia pages, one discovery spurs another. They then quantified their observation with the theoretical concept of “the adjacent possible” proposed by Kaufman [17]. This concept was first proposed in the context of biological evolution and technological evolution [18, 19]. Tria et al. were able to observe counterparts of Heap’s law, whereby the number of discoveries made increases sub-linearly, and Zipf’s law whereby the rank distribution of the frequencies of the discovered elements follow a power-law [16]. These observations were illuminated with a model based on Polya’s urn [20–22] which was able to unify Heap’s and Zipf’s laws and capture the correlations in the discoveries without explicit reference to the unknown space of possibilities to which the concept of “the adjacent possible” refers.

Here we used the PubMed IDs associated with protein-protein interactions (PPIs) as a time-stamp to temporally resolve the discovery dynamics of mammalian and yeast PPINs extracted manually from low-content published studies. We observe the counterparts of Heap’s and Zipf’s laws in the discovery of these mammalian and yeast PPINs. Furthermore, we identify individual proteins which exhibit accelerated or decelerated discovery process rates. We then propose an original model which is related to Polya’s urn. The model features “reinforcement”, rich-get-richer type dynamics with “triggering” whereby novel discoveries trigger the possibility for a subset of new discoveries. Our model is the first network discovery model to explicitly incorporate a space of possibilities, which are the basis of Kaufman’s “adjacent possible”, in the form of an underlying network. Our model captures the observed dynamics of PPIN discovery, and provides strong suggestive evidence that research-focus biases exist within the patterned discovery of the yeast and mammalian PPINs.

## Methods

### Construction of the mammalian PPIN

18 different mammalian PPIN datasets and databases were combined (Table 1). To consolidate interactions, mouse identifiers were converted to their human orthologs using Homologene. Interactions without PMIDs and unary interactions were dropped. 134,590 PPIs from publications that reported more than 10 interactions were also excluded from most analyses. Collectively, the mammalian PPIN consists of 50,478 PPIs covering 9384 proteins, extracted from 34,853 publications with a range of discovery time spanning from April 1967 to October 2013. The yeast (*Saccharomyces cerevisiae*) PPIN was downloaded from iRefWeb 4.1 [23] by including only experimental physical interactions, filtering out unary interactions, and excluding from most analyses 82,391 PPIs from publications associated with more than 10 interactions. The yeast PPIN has 9678 PPIs between 3154 proteins, extracted from 6208 publications with a range of discovery time spanning from June 1946 to November 2011.

**Table 1:** Mammalian PPINs resources

PPI databases	PMID	Publication coverage	PPIs	Latest publication time
BIND	12519993	10069	15895	2010 Aug.
BioCarta	NA	1	189	1994 Jun
BioGrid	16381927	22277	131438	2013 Nov.
DIP	10592249	491	873	2004 Feb.
Ewing et al.	17353931	1	3585	2007 Jan.
HPRD	14681466	18515	35433	2010 Aug.
InnateDB	18766178	3028	6052	2011 Jun.
IntAct	14681455	3300	54248	2013 Jun.
KEA	19176546	6790	16193	2010 Jun.
KEGG	18077471	1	7207	2000 Jan.
MINT	17135203	1265	11750	2009 Oct.
MIPS	14681354	170	323	2004 Jan.
PDZBase	15513994	141	234	2003 Jul.
PPID	21516116	1980	2904	2003 May
SNAVI	16099987	1059	1156	2006 Jan.
Stelzl et al.	16169070	1	1560	2005 Sep.
Rual et al.	16189514	1	4225	2005 Oct.
Total	NA	37015	185068	2013 Nov.

### Entropy calculation

We define the entropy of a sequence of discovery times for PPIs involving a given protein, *i* with known degree ${\tilde{k}}_i$ by:

1 $$ S\left({\tilde{k}}_i\right)=-{\displaystyle \sum_{j=1}^{{\tilde{k}}_i}}\frac{f_j}{{\tilde{k}}_i} log\frac{f_j}{{\tilde{k}}_i} $$

Where *f*_*i*_ is the number of discovered PPIs involving protein *i* in the *j*^*th*^ interval of time, where the time intervals are defined by taking the time at which protein *i* was first observed until the final observation in the whole dataset, and dividing into ${\tilde{k}}_i$ equal-sized bins. This entropy measure was also normalized by dividing by the maximum possible entropy $\log \left({\tilde{k}}_i\right)$.

### Random data permutations

In order to compare the entropy and interval distributions to a null distribution based on uniform randomization of the data, we destroyed the original data order while preserving the frequency distributions by employing random permutations. The first reshuffling method acts globally in time by randomly reassigning the time index to PPI discoveries. The second reshuffling method is local in that it only randomly reassigns time indices from the first appearance of the protein under consideration.

### Generation of artificial networks for the network discovery model

Underlying networks for the PPI discovery model were generated by five different algorithms which resulted in networks with various global properties. In order to approximate the size of the true underlying mammalian PPIN, we constructed artificial networks with 25,000 nodes and tuned the parameters of the different network construction models to produce networks that have ~650,000 links. These numbers agree with a recent estimate of the size of the human PPIN [24].

For creating these background networks, 1) the Barabási-Albert (BA) scale-free network was created using the Barabási-Albert preferential attachment model [25]; 2) the BA cluster network was created using Holme and Kim algorithm [26], which adds an extra step to the Barabási-Albert preferential attachment model, a probability of 0.995 was used to add a link to a node neighbor, so that the average clustering coefficient is close to the observed for the mammalian LC-PPIN; the 3) duplication-divergence (DD) network was generated using the algorithm by Ispolatov et al. [27] with the link retention probability of 0.6473; the 4) Erdős-Rényi random network was created using the algorithm by Batagelj and Brandes [28] with the probability of link creation of 0.00208. The global properties of the underlying networks are summarized in Table 2.

**Table 2:** Properties of the artificial network models

Networks	Nodes	Edges	Clustering coefficient	Power-law exponent	Connected components
BA graph	25000	649324	0.011	1.9	1
BA cluster graph	25000	649304	0.182	2	1
Duplication-Divergence	25000	655271	0	1.7	1
Erdős-Rényi	25000	650069	0.002	NA	1
Complete graph	1000	499500	1	NA	1

### A model of protein-protein interaction network discovery

The true underlying PPIN is represented by the graph *G*(*V*, *E*) where the vertices *V* correspond to the set of all proteins and the edges *E* correspond to the set of all true PPIs. We examine five different network structures in order to study their effect on network discovery dynamics as described above. For a given PPIN, edges are “discovered” by a random choice. At a given time step, the probability of discovering the true link between vertices *i* and *j* is given by, *μ*_*ij*_ ∝ *μ* (${\tilde{k}}_i,{\tilde{k}}_j$), where ${\tilde{k}}_x$ is the currently known degree of vertex *x*. The form of the function *μ* determines the nature of the discovery process in this model, for example,

2 $$ \mathrm{m}\mathrm{u}\left(\mathrm{k}\mathrm{i},,,\mathrm{k}\mathrm{j}\right)\propto Constant $$

corresponds to a uniform unbiased discovery of the network in which all true edges are equally likely to be discovered. A biased PPIN discovery process can be modeled simply by:

3 $$ \mathrm{m}\mathrm{u}\left(\mathrm{k}\mathrm{i},,,\mathrm{k}\mathrm{j}\right)\propto 1+{\tilde{k}}_i+{\tilde{k}}_j $$

In this case there is a process of reinforcement whereby proteins which have many discovered interactions are more likely to be examined for more interactions. Furthermore, we can enhance, what is referred to in Tria et al. [16] as “triggering”, whereby a new discovery triggers adjacent possibilities for subsequent discovery, simply by setting,

4 $$ \mathrm{m}\mathrm{u}\left(\mathrm{k}\mathrm{i},,,\mathrm{k}\mathrm{j}\right)\propto {\tilde{k}}_i+{\tilde{k}}_j $$

In this case only links which are connected to at least one previously discovered protein can possibly become discovered.

In the unbiased case, at times which are far from saturation we expect that the known degree of each protein will increase linearly at a rate which is proportional to its true degree:

5 $$ {\tilde{k}}_i(t) = \frac{d_i}{2{\displaystyle {\sum}_i}{d}_i}t $$

Where *d*_*i*_ is the true of degree i, and the factor of 2 arises because each link is shared by two nodes. In this case we do not expect any significant acceleration of growth for the nodes, i.e., we expect to discover interactions involving any given protein at a roughly constant rate.

### Community structure analysis

The community structure detection algorithm used is based on modularity optimization [29]. The modularity of a partition of community structures measures the density of links inside the communities as compared to links between communities and is defined as [30]:

6 $$ Q = \frac{1}{2m}{\displaystyle \sum_{i,\ j}}\left[{a}_{ij} - \frac{d_i{d}_j}{2m}\ \right]\delta \left({c}_i,{c}_j\right) $$

Where *c*_*i*_ is the community to which node *i* is assigned, $m = \frac{1}{2}{\displaystyle \sum_{ij}}{a}_{ij}$, and *δ*-function *δ*(*u*, *v*) is 1 if *u* = *v* and 0 otherwise, *a*_*ij*_ denote the element of the symmetric adjacency matrix *A* of the graph *G*, and *d*_*i*_, *d*_*j*_ are the degrees of node *i*, *j*, respectively. This unsupervised algorithm involves modularity optimization by local changes to communities and aggregation of communities to build new communities. As a result, the algorithm generates a hierarchy of community structures. In practice, a Python implementation named “python-louvain” of this algorithm was applied.

## Results

**Fig. 1:**
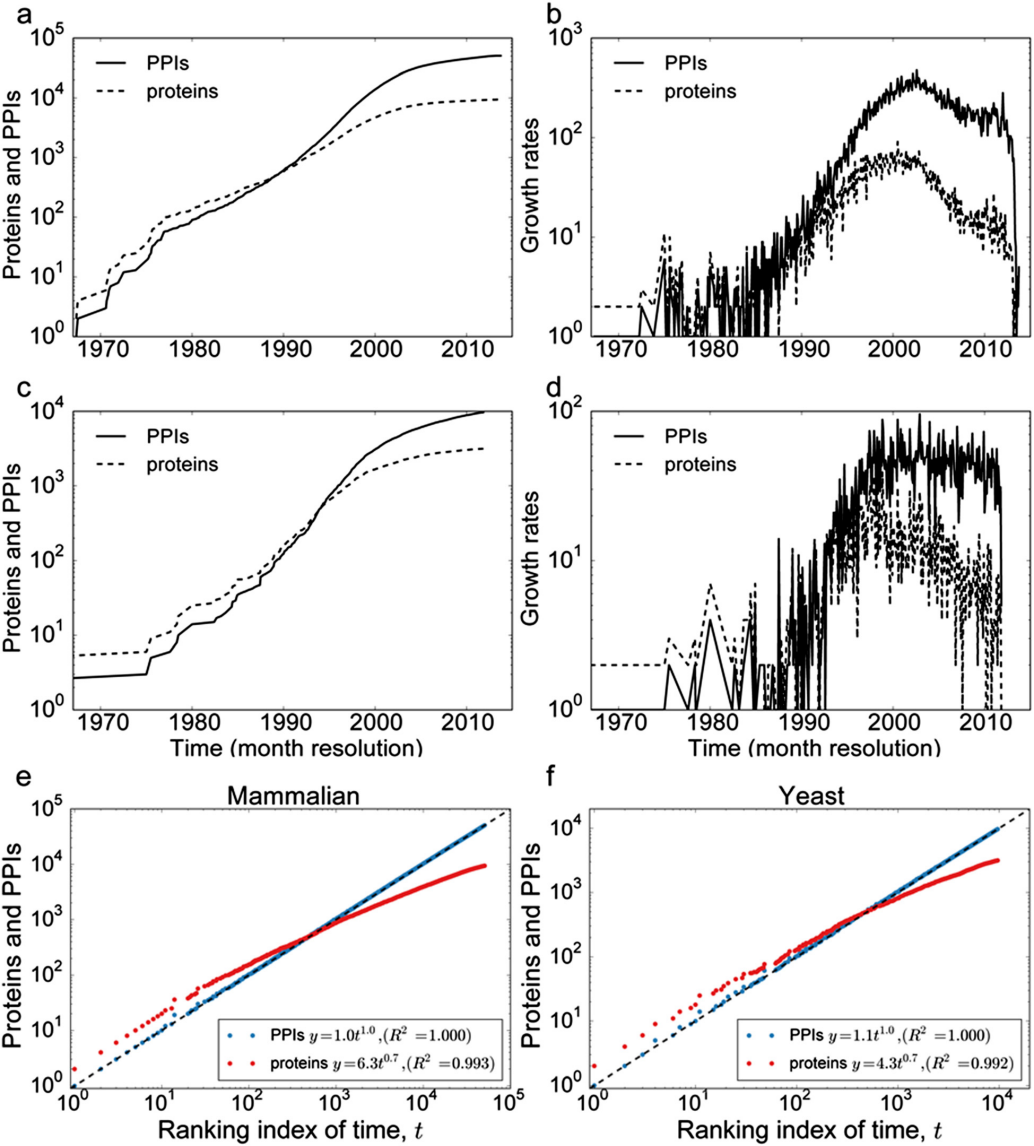
Discovery of the mammalian and yeast LC-PPINs over time. Accumulation of discovered proteins (dotted line) and their interactions (solid line) and the discovery rate of interactions and proteins in the mammalian (**a**, **b**) and yeast (**c**, **d**) literature based PPINs. The accumulation of discovered proteins (red dots) and their interactions (blue dots) are plotted with respect to the ranking index of time for mammalian (**e**) and yeast (**f**) PPINs

The number of unique mammalian PPIs and proteins discovered each month, as well as the rate of discovery has few modes (Fig. 1a-d). In order to eliminate extrinsic factors, such as the changing pace of scientific discovery, while retaining the intrinsic properties of the PPINs discovery process, we converted the real-time discovery of each PPI to a time-ranked order. The discovery process of unique proteins appears to be sub-linear, which is analogous to Heap’s law, which states that the number of unique words increases sub-linearly with the length of text (Fig. 1e-f).

**Fig. 2:**
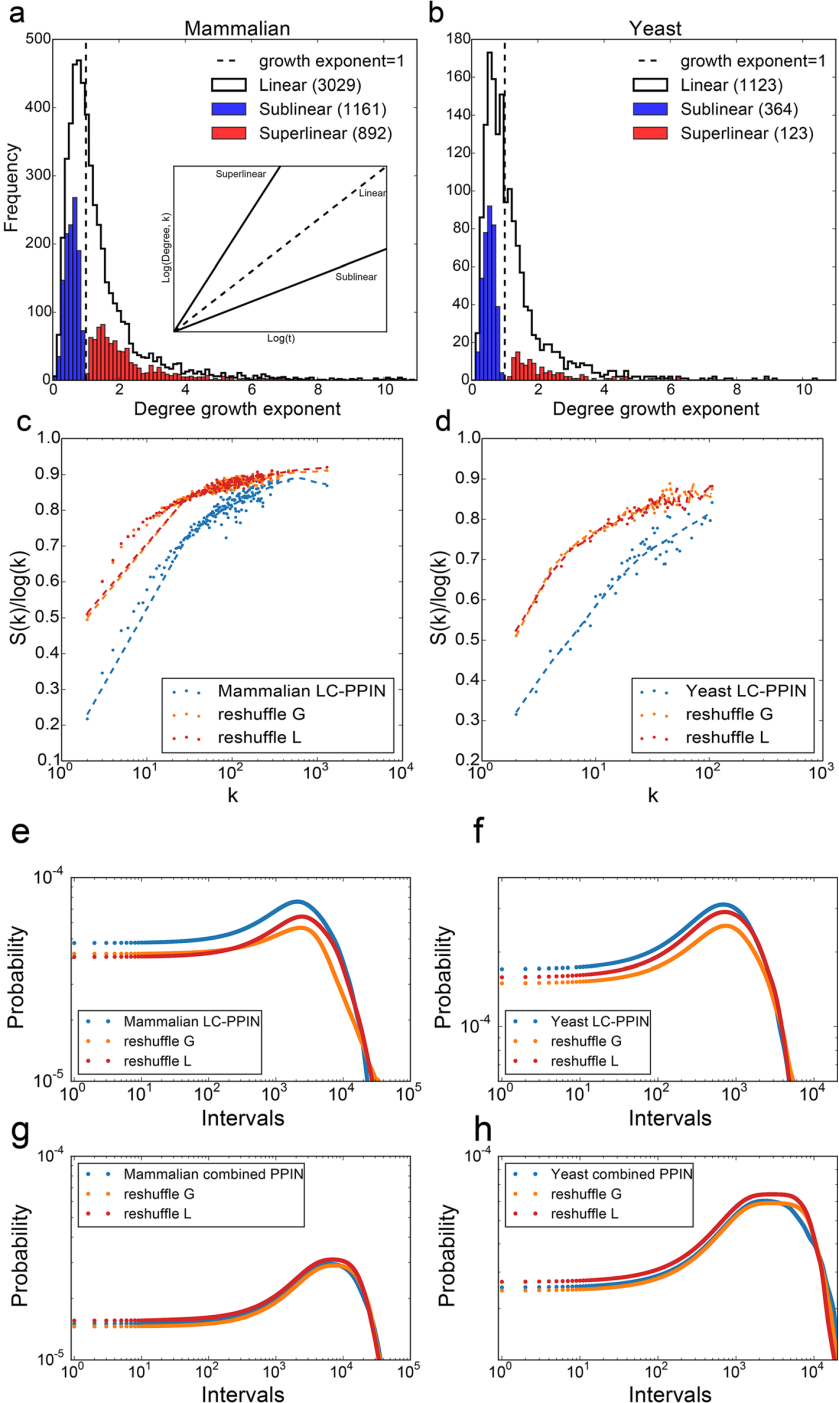
The dynamics of individual proteins in the discovery of mammalian and yeast LC - and combined PPINs. **a**-**b** The distribution of growth exponents of the degrees of individual proteins; super-linear growth corresponds to an acceleration in the rate of discovery of PPIs involving the protein in question. **c**-**d** The normalized entropy plotted against the mean degree of the actual PPI discovery for the real network and also for reshuffled versions. **e**-**h** The distribution of time intervals between PPI discoveries involving each protein for the real PPI discovery process and also randomly reshuffled data in LC-PPINs (**e**-**f**) and combined PPINs made from both high-content and low-content studies (**g**-**h**)

In addition to the global properties of the discovered network, it is also important to examine local dynamical properties, such as the degree of individual proteins as a function of time. We observe that most proteins increase in degree linearly in both mammalian and yeast networks (Fig. 2a-b). Notably, many proteins are growing in their degree super-linearly. This super-linearity corresponds to acceleration in the rate at which publications are reporting interactions involving the protein. Examples of proteins with super- and sub- linear degree growth are shown in Additional file 1: Figure S1.

To examine these possibilities we compared the observed distribution of proteins with accelerated or decelerated rates to the distributions observed for random permutations of the same data (Fig. 2c-f). Similar null distributions were also examined by Tria et al. [16] in a completely different context. This analysis shows that there are significantly more proteins that are growing super-linearly than would be expected by random chance. This is indicative of correlations in the discovery process of PPIs – discoveries involving particular proteins tend to arrive in bursts with their corresponding short time intervals. To explore whether the correlated discovery of PPIs is a unique property of the low-content PPINs, we constructed mammalian and yeast PPINs by increasing the threshold for the maximum number of PPIs per publication from 10 to 50, to 100, to 1000 and with no threshold/filter at all. Observing the distribution of the discovery intervals for PPIs, we see that after including the high content studies, the distribution of intervals is similar to the distribution for randomly permuted data (Fig. 2g-h and Additional file 2: Figure S2). Interestingly, the entropy measure still shows difference between randomly shuffled discoveries and networks discovered by low- and high-content methods combined. We believe that this may be an artifact of the sparse data from high content PPIs, or a new type of bias within PPI data collected by high content methods. For example, PPIs from mass-spectrometry proteomics are known to be biased in detecting large, abundant or sticky proteins.

In principle, all parts of a PPIN are discoverable and a uniform exploration is theoretically possible. However, in practice, the discovery process appears to be correlated. In order to illuminate the dynamics of PPINs discovery we introduce a simple model. With reference to Kaufman’s “expanding the adjacent possible” [17] we explicitly incorporate the space of possibilities in the form of an underlying true network. We begin with a random uniform exploration process, and then by modulating the probability of discovering links based on the already discovered network, we study the effect research focus biases can have on the dynamics of the network discovery process. A schematic representation of this model is shown in Additional file 3: Figure S3. Although, the true PPIN is unknown, we can examine the effect of global network properties within this model.

**Fig. 3:**
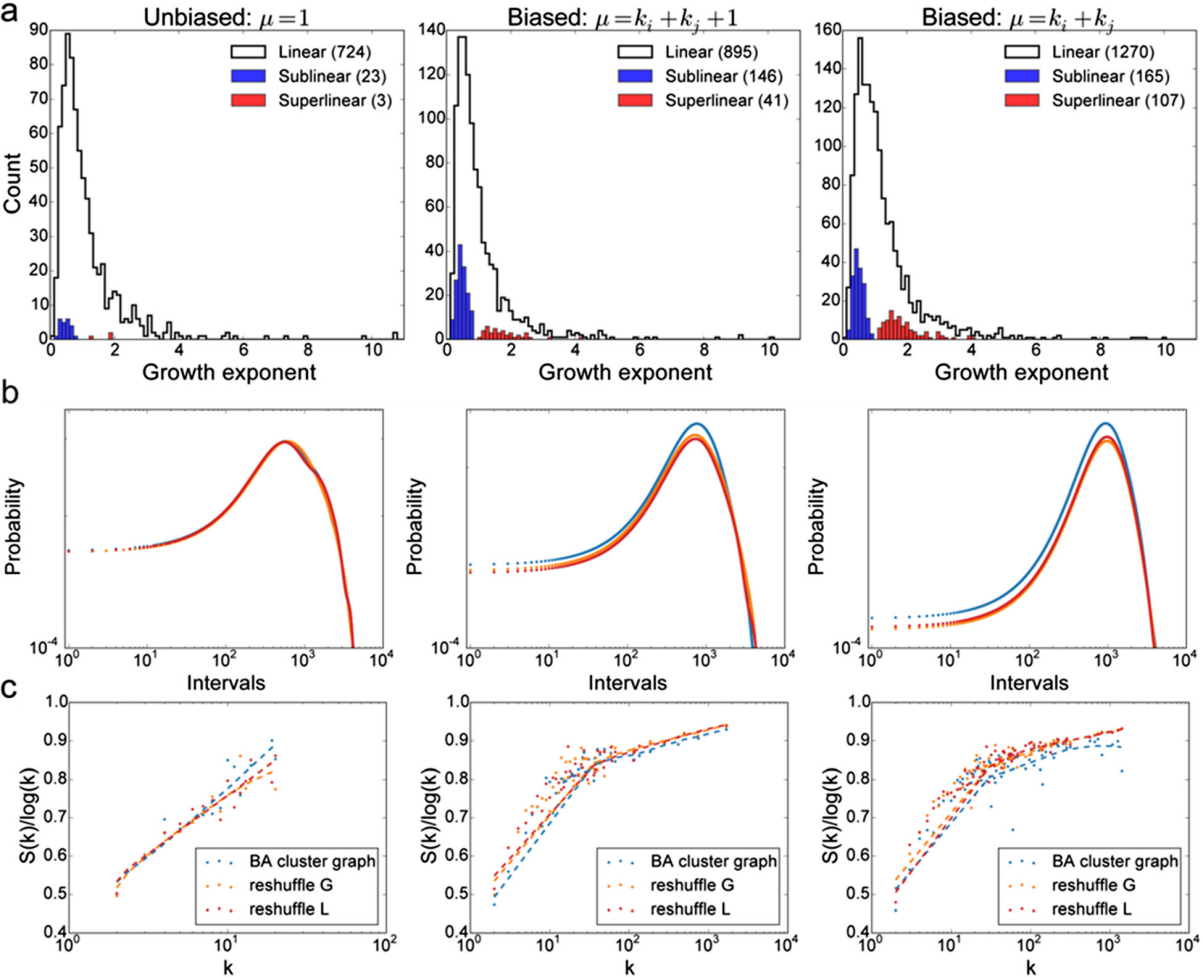
Three model realizations with the scale-free (BA) clustered underlying artificial PPIN. **a** Distribution of degree growth exponents; (**b**) distribution of time intervals between PPI discoveries involving each protein; (**c**) the normalized entropy of PPI discoveries for each protein averaged over each degree

When we examine the distribution of the growth exponent of the degrees of each node in the model, we see that highly accelerating nodes only occur in the biased models, and the effect of including triggering enhances this effect (Fig. 3). These results are for the scale-free (BA) clustered artificial network as the underlying network; for the other artificial network models these results vary (Additional file 4: Figure S4, Additional file 5: Figure S5, Additional file 6: Figure S6, Additional file 7: Figure S7).

Furthermore, we notice that accelerating nodes only occur in the models where the underlying networks have a power-law degree property (Additional file 8: Figure S8). This illustrates the relevance of the underlying network structure. It seems that the topology of the space of possibilities has an impact on the discovery process. We note that the difference between the biased and unbiased models is not as marked as the real PPI discovery (Additional file 8: Figure S8). However, it is clear that network discovery of the real networks must contain biases.

**Fig. 4:**
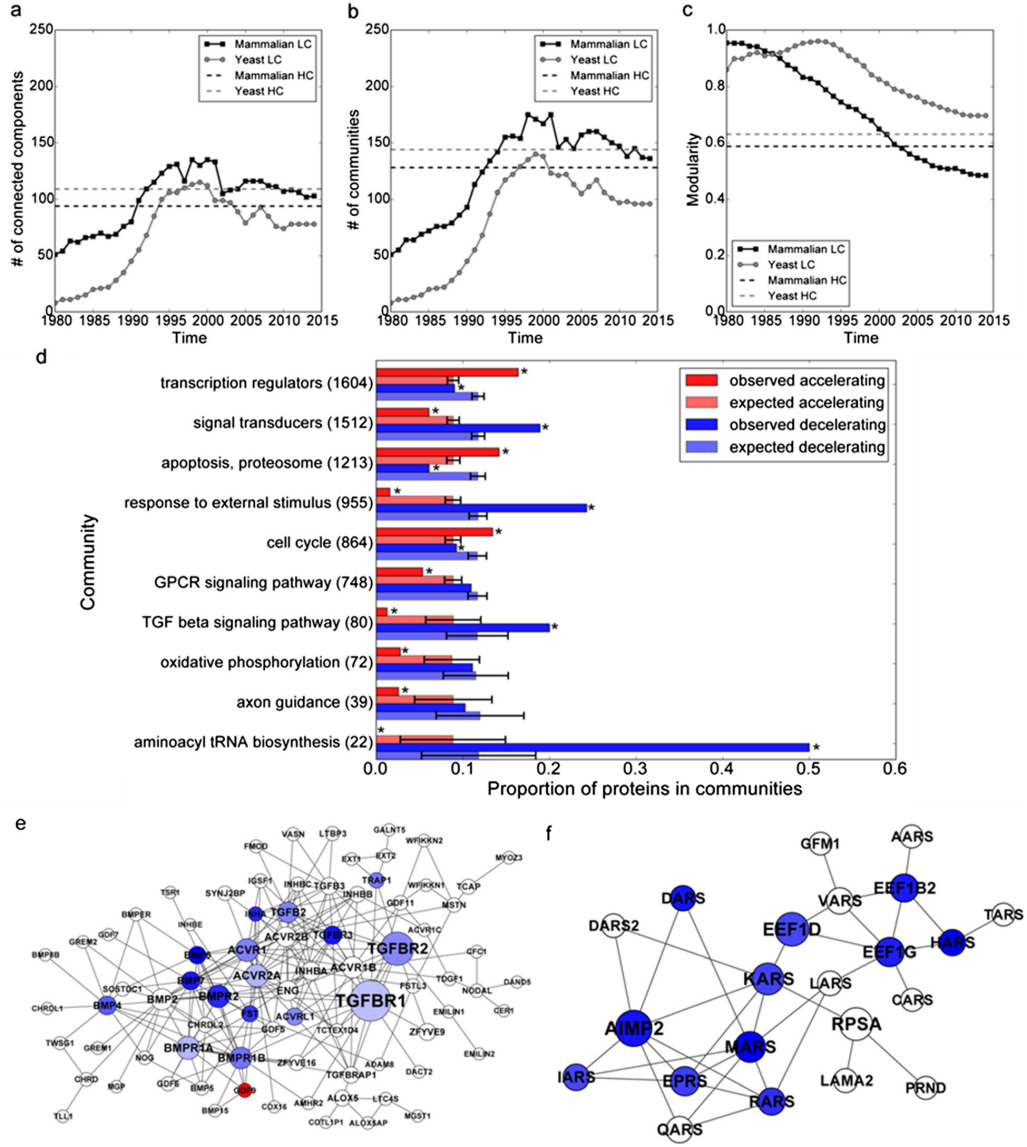
Relationship between community structure and PPI discovery rates in PPINs. **a** Connected components; (**b**) Communities; (**c**) modularity, which a quantity that measures the strength of community partition compared to random [30]. **d** Clusters with significant over-representation of proteins with accelerating or decelerating PPI discovery rates. **e**, **f** Subnetworks connecting proteins from two representative cold clusters where proteins are connected through their known interactions with other members of the cluster

Our ability to mark individual proteins as either accelerating or decelerating in their discovery rates can be used to identify hot and cold discovery regions within the mammalian PPIN. For identifying such regions, we applied a network clustering algorithm to decompose the networks into clusters, and then computed the average discovery rate within each cluster (Fig. 4). As expected, out of 102 clusters identified, several clusters are enriched for rapidly accelerated or decelerated proteins within each cluster. Each cluster with significant enrichment for accelerating or decelerating rates is labeled by its most significant gene ontology enriched term (Fig. 4d). The network contains two notable clusters with decelerating discovery rates: TGF beta signaling (Fig. 4e) and aminoacyl tRNA biosynthesis (Fig. 4f).

## Discussion

By time-resolving the mammalian and yeast literature-based PPINs we identified a clear pattern in the PPI discovery process. This pattern is consistent with a biased discovery process which exhibits properties of reinforcement, whereby commonly studied proteins are more likely to be further studied in the near future, and with triggering, whereby discoveries spur related discoveries in the PPI network neighborhood. We introduced a model of PPI network discovery which supports the idea that research focus bias is relevant in the discovery process of mammalian and yeast PPIs. The model demonstrates that network discovery can explain the existence of many more proteins whose degree is accelerating compared with the number of such proteins in more random discovery processes. Such trends should be considered when reusing PPI data for interpretation of new results for drawing conclusions about the underlying biology, and for making decisions about the next set of experiments. A recent publication by Schnoes et al. [31] suggested that there exist significant biases in the discovery of gene functional annotations, and this has a significant effect on their interpretation and application to biological investigations, here we extended this observation to the discovery of PPIs.

Our model of PPI network discovery also revealed that an underlying network with the scale-free property is also necessary for the appearance of proteins with super-linear degree growth, which supports the hypothesis that the topology of the real PPINs is scale free [25, 32, 33]. Interestingly, the local clustering of the underlying network does not seem to play a role in the emergence of biases during the discovery process. Notably, the observed bias is stronger in mammalian than yeast PPINs in terms of the ratio of proteins with super-linear degree growth. One explanation for this is that the discovered mammalian PPIN is further from saturation compared to yeast, which is supported by the estimated size of human and yeast PPINs [24]. To explore whether the effects of research focus bias introduced in low-content studies can be reduced, we included PPIs from high-throughput studies. We observed the overall reinforcement and triggering effects on the discovery process are mitigated. However, those effects can still be revealed on the discovery of PPIs for many individual proteins (Additional file 2: Figure S2), suggesting the inclusion of high-content studies help to some extent to reduce the research focus bias in LC-PPINs.

## Conclusions

Recent studies demonstrate that experimental methods that identify many reliable PPIs in a single study show more uniform distribution of PPIs [3, 34]. However, current high cost, requirement for specific skills, and years of concentrated efforts, are still great obstacles toward making such profiling experiments more widely applied and accepted. In principle, the shift toward genome-wide system-level biology is expected to correct and better inform our current understanding of the real PPINs. In addition, the view of binary PPI is limited. It is now well established that most proteins within cells work as a part of macro-molecular complexes, and thus we expect that the in-silico reconstruction of such complexes will become more central, while less emphasis will be placed on the identification and reuse of binary PPIs. Nevertheless, methods that correct for research focus biases can potentially improve the use of such PPIN and pathway databases for their various computational applications.

## Additional files

Additional file 1: Figure S1. The time-dependence of protein degrees for (A, C) hub proteins; (B, E) accelerating proteins; and (C,F) decelerating proteins of mammalian (A-C) and yeast (D-F) LC-PPINs. The degree growth exponents (slopes) are indicated in the legends. Additional file 2: Figure S2. The dynamic of network discovery of mammalian and yeast PPINs made from different PPI per publication cutoffs. Normalized entropy of PPI discoveries for each protein averaged over each degree as well as the distribution of the time intervals between PPI discoveries involving each protein are plotted for each network. The numbers of PPIs per publication cutoff used for construction of each network are indicated at the top of each column. Additional file 3: Figure S3. Schematic of three realizations of the network discovery model. The same graph serves as the underlying, true PPI network in each case. Nodes in the graph correspond to proteins and edges correspond to PPIs. Edges are “discovered” randomly and the discovery is indicated red. In the unbiased model each edge is equally likely to be discovered. In the model realization with reinforcement the probability of discovering an edge is proportional to the sum of the degrees of the proteins it connects such that edges connecting higher degree proteins are more likely to be discovered as indicated by the weight of the edge line. In the last example, the triggering process in involved, whereby new discoveries open-up the possibility of further discoveries; in this model only edges which are connected to a discovered protein are discoverable while also the reinforcement property is maintained. Additional file 4: Figure S4. Three model realizations with a BA graph as underlying the PPIN. (A) Distribution of degree growth exponents; (B) distribution of the time intervals between PPI discoveries involving each protein; (C) normalized entropy of PPI discoveries for each protein averaged over each degree. Additional file 5: Figure S5. Three model realizations with a duplication-divergence graph as the underlying PPIN. (A) Distribution of degree growth exponents; (B) Distribution of the time intervals between PPI discoveries involving each protein; (C) normalized entropy of PPI discoveries for each protein averaged over each degree. Additional file 6: Figure S6. Three model realizations with a Erdős-Rényi random graph as the underlying PPIN. (A) Distribution of degree growth exponents; (B) Distribution of the time intervals between PPI discoveries involving each protein; (C) normalized entropy of PPI discoveries for each protein averaged over each degree. Additional file 7: Figure S7. Three model realizations with a complete graph as the underlying PPIN. (A) Distribution of degree growth exponents; (B) Distribution of the time intervals between PPI discoveries involving each protein; (C) normalized entropy of PPI discoveries for each protein averaged over each degree. Additional file 8: Figure S8. Ratios of proteins in actual and model realizations of PPINs with super-linear and sub-linear growth of PPIs. Each model realization was performed three times and standard deviations of the ratios are indicated by the error bars.
