# Supplementary figures and images for: Dynamics of the discovery process of protein-protein interactions from low content studies
Source: BMC Syst Biol. 2015 Jun 6;9:26. doi: 10.1186/s12918-015-0173-z (PMC4456804; doi:10.1186/s12918-015-0173-z)

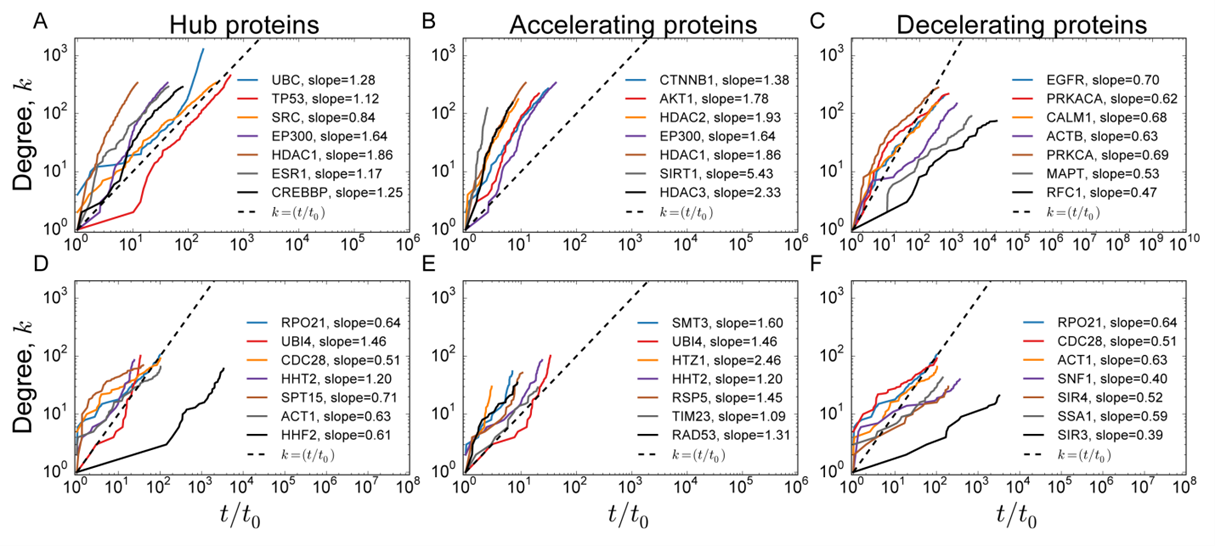

Supplement: Additional file 1: Figure S1. — The time-dependence of protein degrees for (A, C) hub proteins; (B, E) accelerating proteins; and (C,F) decelerating proteins of mammalian (A-C) and yeast (D-F) LC-PPINs. The degree growth exponents (slopes) are indicated in the legends. [file 12918_2015_173_MOESM1_ESM.png]

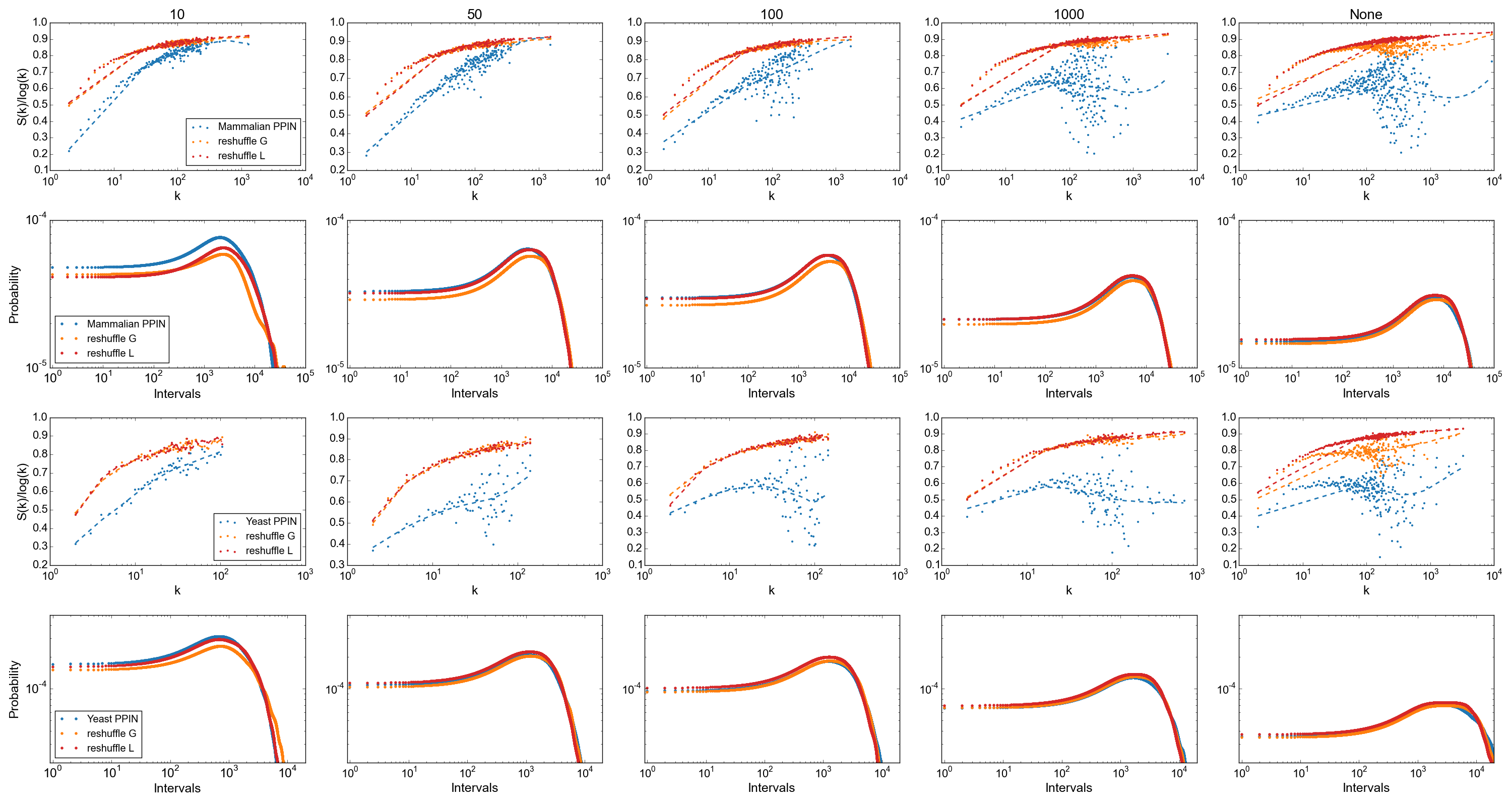

Supplement: Additional file 2: Figure S2. — The dynamic of network discovery of mammalian and yeast PPINs made from different PPI per publication cutoffs. Normalized entropy of PPI discoveries for each protein averaged over each degree as well as the distribution of the time intervals between PPI discoveries involving each protein are plotted for each network. The numbers of PPIs per publication cutoff used for construction of each network are indicated at the top of each column. [file 12918_2015_173_MOESM2_ESM.png]

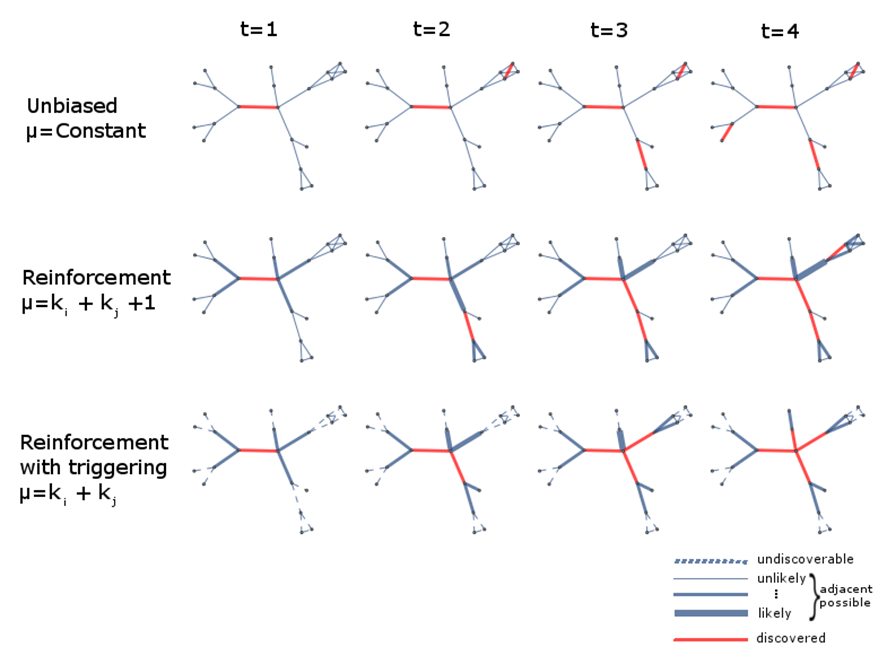

Supplement: Additional file 3: Figure S3. — Schematic of three realizations of the network discovery model. The same graph serves as the underlying, true PPI network in each case. Nodes in the graph correspond to proteins and edges correspond to PPIs. Edges are “discovered” randomly and the discovery is indicated red. In the unbiased model each edge is equally likely to be discovered. In the model realization with reinforcement the probability of discovering an edge is proportional to the sum of the degrees of the proteins it connects such that edges connecting higher degree proteins are more likely to be discovered as indicated by the weight of the edge line. In the last example, the triggering process in involved, whereby new discoveries open-up the possibility of further discoveries; in this model only edges which are connected to a discovered protein are discoverable while also the reinforcement property is maintained. [file 12918_2015_173_MOESM3_ESM.png]

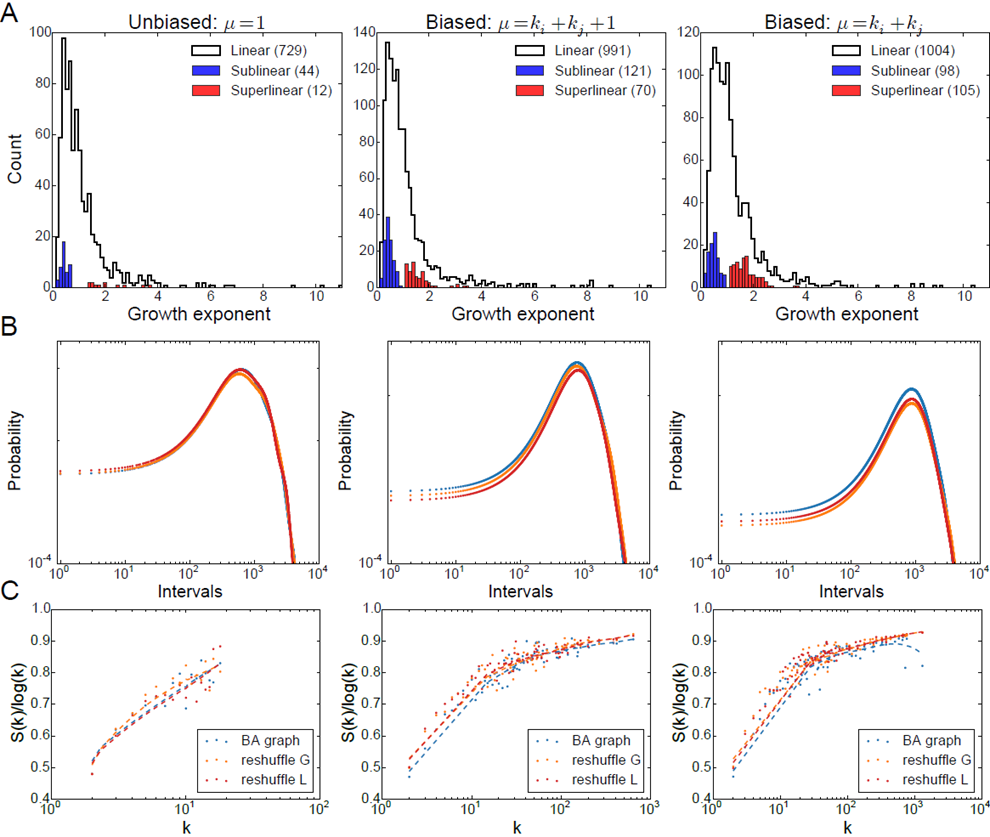

Supplement: Additional file 4: Figure S4. — Three model realizations with a BA graph as underlying the PPIN. (A) Distribution of degree growth exponents; (B) distribution of the time intervals between PPI discoveries involving each protein; (C) normalized entropy of PPI discoveries for each protein averaged over each degree. [file 12918_2015_173_MOESM4_ESM.png]

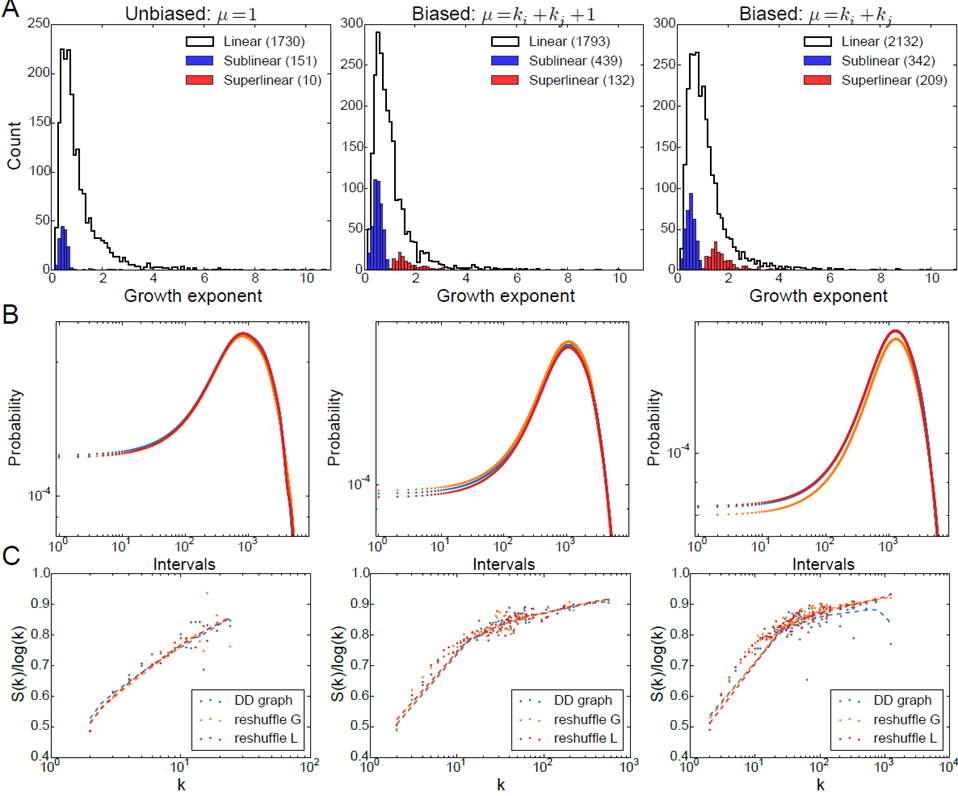

Supplement: Additional file 5: Figure S5. — Three model realizations with a duplication-divergence graph as the underlying PPIN. (A) Distribution of degree growth exponents; (B) Distribution of the time intervals between PPI discoveries involving each protein; (C) normalized entropy of PPI discoveries for each protein averaged over each degree. [file 12918_2015_173_MOESM5_ESM.png]

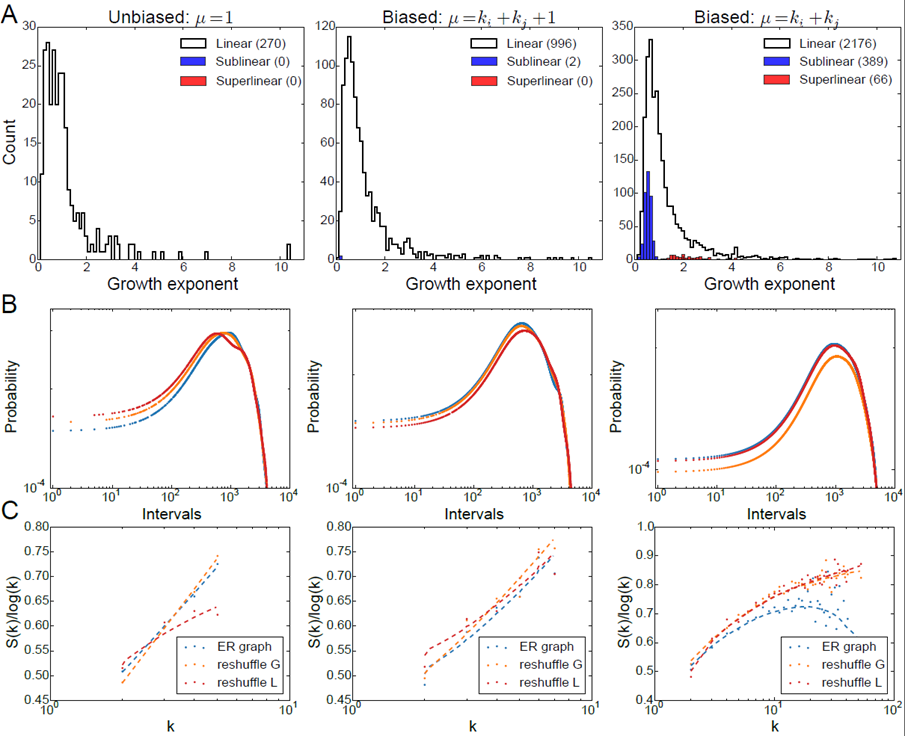

Supplement: Additional file 6: Figure S6. — Three model realizations with a Erdős-Rényi random graph as the underlying PPIN. (A) Distribution of degree growth exponents; (B) Distribution of the time intervals between PPI discoveries involving each protein; (C) normalized entropy of PPI discoveries for each protein averaged over each degree. [file 12918_2015_173_MOESM6_ESM.png]

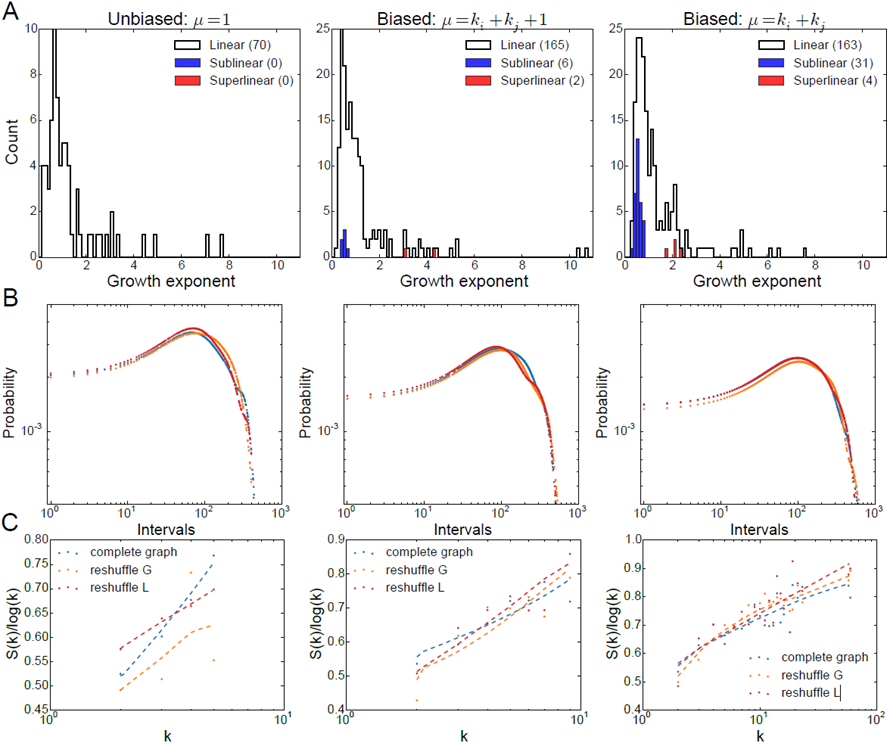

Supplement: Additional file 7: Figure S7. — Three model realizations with a complete graph as the underlying PPIN. (A) Distribution of degree growth exponents; (B) Distribution of the time intervals between PPI discoveries involving each protein; (C) normalized entropy of PPI discoveries for each protein averaged over each degree. [file 12918_2015_173_MOESM7_ESM.png]

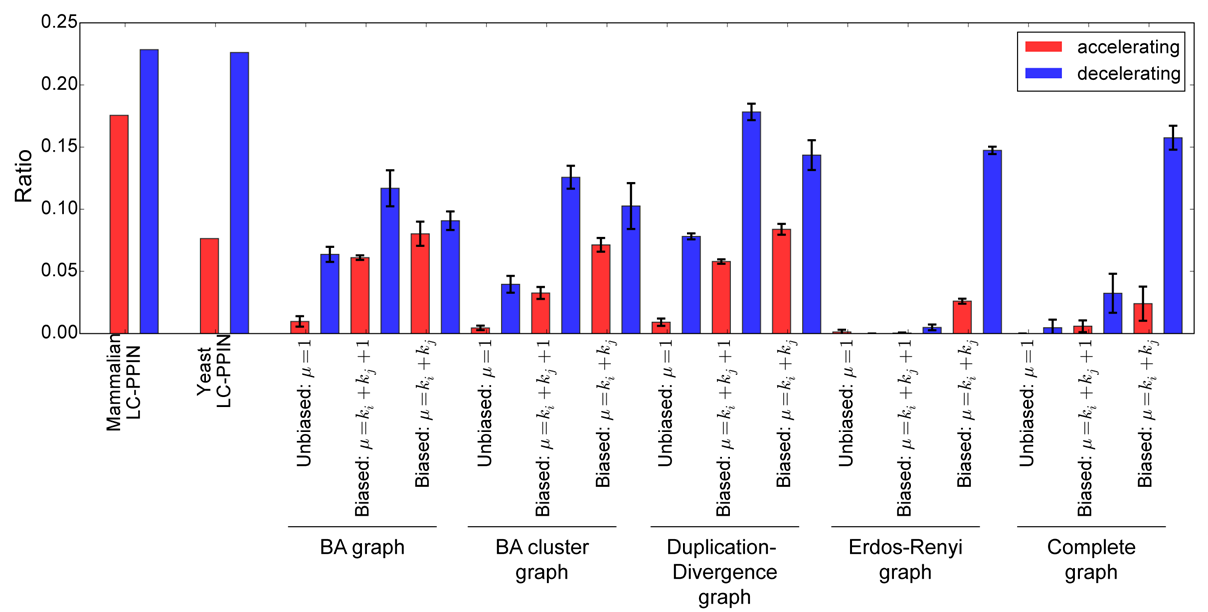

Supplement: Additional file 8: Figure S8. — Ratios of proteins in actual and model realizations of PPINs with super-linear and sub-linear growth of PPIs. Each model realization was performed three times and standard deviations of the ratios are indicated by the error bars. [file 12918_2015_173_MOESM8_ESM.png]
